# Machine learning for predicting CKD stages in patients with autosomal dominant polycystic kidney disease: a nationwide cohort study in Japan

**DOI:** 10.1038/s41598-026-39885-7

**Published:** 2026-02-13

**Authors:** Yosuke Shimada, Hiroshi Kataoka, Saori Nishio, Junichi Hoshino, Keiju Hiromura, Yoshitaka Isaka, Satoru Muto

**Affiliations:** 1 Intelligent Systems Laboratory, SECOM CO., LTD., Tokyo, Japan; 2 Department of Infection Control Science, Juntendo University Graduate School of Medicine, Tokyo, Japan; 3 Department of Nephrology, Tokyo Women’s Medical University, Tokyo, Japan; 4 Department of Hemodialysis and Apheresis, Hokkaido University Hospital, Hokkaido, Japan; 5 Department of Nephrology and Rheumatology, Gunma University Graduate School of Medicine, Gunma, Japan; 6 Department of Nephrology, Graduate School of Medicine, The University of Osaka, Osaka, Japan; 7 Department of Urology, Juntendo University Nerima Hospital, Tokyo, Japan

**Keywords:** Machine learning, ADPKD, Chronic diseases, Nonlinear relationships, Random forest, Personalized medicine, Computational biology and bioinformatics, Diseases, Medical research, Nephrology

## Abstract

Machine learning (ML) is a valuable tool in healthcare, enabling the prediction of disease progression through data-driven regression and nonlinear modeling. Unlike traditional statistical methods, ML can identify complex interactions among explanatory variables. Autosomal dominant polycystic kidney disease (ADPKD) is a common cause of chronic kidney disease (CKD), often progressing to end-stage renal failure. Accurately predicting CKD progression in ADPKD patients is essential for personalized treatment strategies. This study analyzed data from 2,737 patients with ADPKD enrolled in the Japanese Nationwide Cohort. Using this dataset, we developed ML models to predict CKD stages. Feature importance analysis was performed to identify key predictive variables. Three ML models—random forest, support vector machine, and naïve Bayes—were evaluated for their predictive accuracy. Random forest exhibited the highest predictive accuracy among the models tested. Feature importance analysis identified estimated glomerular filtration rate (eGFR), serum creatinine, CKD heat map, urinary protein, and total kidney volume as the most significant predictors of CKD stage. As a nonlinear model, random forest effectively captured complex interactions between variables, outperforming the linear support vector machine. The naïve Bayes model, despite assuming independence among variables, surpassed the linear model, indicating limited interdependence among some predictors. ML, particularly random forest, provides a robust approach for predicting CKD stages in patients with ADPKD by accounting for nonlinear variable relationships. These findings emphasize ML’s potential in personalized CKD management and highlight the need for individualized treatment approaches.

## Introduction

Machine learning is a valuable tool for modern healthcare, offering data-driven regression with non-linear models to predict target variables^1–4^. Unlike traditional statistics, which use linear models to identify significant risk factors, machine learning can uncover non-linear relationships between explanatory and target variables^2–5^. Although most machine learning models are implicit, some are transparent enough to reveal novel related factors^5^. Machine learning also handles high-dimensional data, using dimensionality reduction techniques^2,3,6^, making it ideal for biological data with many variables. Furthermore, clinicians can leverage transparent machine learning to explore the significance of key variables, making it effective for addressing the complexity and diversity of chronic diseases.

Autosomal dominant polycystic kidney disease (ADPKD) is a common cause of chronic kidney disease (CKD), characterized by the gradual development of kidney cysts and often associated with complications like polycystic liver disease and intracranial aneurysms (IA)^7–9^. Approximately 50% of individuals with ADPKD will develop end-stage renal failure by age 60^10^. Mutations in the *PKD1* and *PKD2* genes are usually central to disease initiation and progression^11–13^, but acquired factors also play an important role^14^, complicating disease management. Accordingly, the development of predictive models utilizing machine learning is expected to facilitate personalized management of ADPKD.

In Japan, the Ministry of Health, Labour, and Welfare offers financial assistance to patients with intractable diseases, including ADPKD^15^. Those receiving support are registered in a national database^15^, allowing researchers to analyze disease trends and evaluate treatment effectiveness^16^. In this study, we aimed to determine the key factors and develop machine learning models to predict future CKD stages in patients with ADPKD using a big data from a Japanese nationwide registry.

Mutations in the PKD1 gene play a central role in diagnosis and prognosis, however, genetic testing for ADPKD is not covered by Japan’s national health insurance. As a result, such testing is rarely performed in routine clinical practice. The PROPKD score is a prognostic model with high predictive performance; nevertheless, because it requires genetic data, its applicability is limited in countries where genetic testing is not routinely available^17^. Several prognostic models that do not rely on genetic data have also been proposed, aiming to achieve high performance by directly using MRI images as input^18,19^. However, in large registries including Japan’s national database for intractable diseases, it is uncommon to store raw MRI images, which poses challenges for validating such approaches in large-scale datasets.

In this study, we developed a prognostic model for ADPKD that does not require genetic testing and instead utilizes data readily available in routine clinical practice. This approach is expected to serve as a practical tool for screening purposes in everyday clinical settings.

## Methods

### Design, setting, and participants

In this retrospective study, we used the National Database of Designated Intractable Diseases of Japan, established under the Intractable Disease Health Care Act in Japan^20^. Japan’s system of medical registries and subsidies for designated intractable diseases has been described previously^20^. Anonymized data based on Medical Certificates of Designated Intractable Diseases of newly enrolled patients between the 2015 and 2021 fiscal years were provided by the Japanese Ministry of Health, Labour and Welfare based on an application submitted for this study. Patients diagnosed with ADPKD who satisfy the intractable disease criteria^9^ apply for medical expense assistance from the government and are registered in the intractable disease database^15, 20^. Criteria for ADPKD designation as an intractable disease in Japan include the following: (1) red area of the CKD severity classification of the Kidney Disease Improving Global Outcomes (KDIGO) heat map (patients with advanced CKD stage or severe proteinuria)^21^ and (2) total kidney volume (TKV) of ≥ 750 mL and increase in TKV of ≥ 5% per year^9^. This study was conducted in accordance with the 1964 Declaration of Helsinki and its later amendments or comparable ethical standards. The study was approved by the institutional ethics boards of the Japanese Society of Nephrology (authorization number: 70) and Juntendo University (authorization number: 2021016), and owing to the data characteristics the requirement for individual consent for this retrospective analysis was waived.

This study enrolled 2,737 patients who completed their initial application during the study period, with the CKD stage G classification (categorized by GFR) evaluated at the time of the renewal application three years later. The database consists of baseline data collected at the time of initial case registration and annual follow‑up data recorded thereafter. Because each follow‑up entry contains only a single measurement obtained by the attending physician, no preprocessing was applied, and the recorded values were used directly as features. Using this data source, a dataset was constructed, in which the clinical characteristics recorded at the initial application were designated as predictors, and the CKD stage determined at the three-year renewal application was defined as the outcome.

### Outcome evaluations

The primary endpoint was the CKD stage G classification assessed three years later. Both the G and A classifications of CKD stage serve as severity criteria for determining eligibility for financial support for intractable diseases in Japan and constitute important indicators that influence clinical management. Among these indicators, the G classification is widely incorporated into mandatory health checkups and is frequently measured in routine clinical practice, making it advantageous as ground‑truth data for machine‑learning analyses.

### Statistical analyses

To assess the distribution of the data, descriptive statistics were computed for all predictors. Discrete variables were summarized by frequency and proportion, while continuous variables were characterized by mean and variance. Additionally, the number of observations was recorded for each variable. All analyses were conducted using R version 4.4.2.

### Model fitting and evaluation

A classification model was developed to predict CKD stage three years after initial application, using clinical characteristics at the first time of application as input variables. The machine learning methods considered included the Naive Bayes model, linear support vector machine, and random forest. Stratified sampling was performed according to the three-year outcome of CKD stage G classification, after which patients were assigned to the training set (60%) for parameter optimization and the testing set (40%) for external validation. The training set was utilized to generate multiple samples through k-fold cross-validation, enabling the identification of parameters that demonstrated stable performance across diverse datasets. The final model’s accuracy and ROC-AUC were calculated using the testing set, which was not involved in parameter optimization, to assess its predictive performance. ROC-AUC, representing the area under the receiver operating characteristic curve, serves as an indicator of the model’s capacity to assign higher probabilities to correct labels.

Hyperparameter tuning in training set was performed using grid search, with five candidate values defined for each parameter. For the random forest model, the grid included the number of explanatory variables used in each tree (mtry: {4, 19, 35, 50, 66}), the number of trees (trees: {1, 500, 1000, 1500, 2000}), and the minimum number of observations required in a terminal node (min_n: {2, 11, 21, 30, 40}), resulting in 125 hyperparameter combinations (5 candidate values for each of the three parameters). For the linear support vector machine, the grid consisted of the cost parameter controlling the penalty for misclassification (cost: {0.000977, 0.0131, 0.177, 2.38, 32.0}) and the margin parameter defining the distance to the separating hyperplane (margin: {0.00, 0.05, 0.10, 0.15, 0.20}). For the naive Bayes model, the grid included the smoothness parameter for probability density estimation (smoothness: {0.50, 0.75, 1.00, 1.25, 1.50}), the Laplace smoothing factor (laplace: {0.00, 0.75, 1.50, 2.25, 3.00}), and the threshold for the correlation coefficient used in feature selection (threshold: {0.00, 0.25, 0.50, 0.75, 1.00}).

When constructing the model with the three machine learning methods, preprocessing was tailored to the specific characteristics of each approach. Although the Naive Bayes model is capable of handling datasets with missing values, it is known that high correlations between predictor variables can adversely affect predictive performance. Consequently, in cases where variables exhibited high correlation coefficients, one of the correlated variables was excluded during preprocessing. In contrast, since the Support Vector Machine and Random Forest methods are unable to process missing values, continuous variables were imputed using mean values, and discrete variables were imputed using mode values during preprocessing. Furthermore, it is known that the predictive performance of the Naive Bayes model decreases when there is a correlation between predictor variables. Therefore, in cases where high correlation coefficients were observed, one of the correlated variables was excluded from the model.

To observe the constructed model and assess the contribution of each feature to its predictions, permutation feature importance was calculated. This method quantifies feature importance by measuring the increase in prediction error after randomly shuffling the values of a specific feature.

## Results

Table 1 presents the aggregated clinical characteristics in the dataset.

**Table 1:** Statistical analysis of baseline clinical characteristics (explanatory variable).

Variable	Overall	G1	G2	G3a	G3b	G4	G5
ALT	18.4 ± 15.5 [2105]	22.0 ± 16.2 [43]	18.7 ± 11.8 [276]	19.7 ± 11.1 [320]	20.1 ± 15.8 [430]	19.4 ± 21.5 [515]	14.8 ± 11.1 [521]
AST	20.7 ± 10.1 [2106]	22.6 ± 16.5 [43]	19.8 ± 7.6 [276]	21.0 ± 6.5 [319]	21.9 ± 8.6 [429]	21.1 ± 9.7 [515]	19.2 ± 13.3 [524]
CT, MRI: Number of cysts in each kidney	38.1 ± 38.8 [407]	24.7 ± 14.3 [13]	34.5 ± 45.2 [49]	36.6 ± 37.0 [52]	31.8 ± 23.4 [84]	41.1 ± 36.9 [107]	44.4 ± 48.7 [102]
Number of cysts Left	41.3 ± 31.0 [43]	[< 10]	[< 10]	[< 10]	[< 10]	46.0 ± 36.0 [12]	48.2 ± 35.7 [12]
Number of cysts Right	43.5 ± 38.5 [43]	[< 10]	[< 10]	[< 10]	[< 10]	46.0 ± 36.0 [12]	46.9 ± 35.2 [12]
Kidney volume growth rate	11.8 ± 17.1 [1823]	18.1 ± 20.3 [38]	12.4 ± 17.6 [276]	11.8 ± 15.5 [302]	13.0 ± 25.2 [396]	10.8 ± 10.3 [420]	10.7 ± 12.9 [391]
Diastolic blood pressure	83.0 ± 13.6 [2520]	80.3 ± 12.0 [46]	81.9 ± 12.3 [294]	83.5 ± 13.2 [343]	84.1 ± 12.9 [459]	83.3 ± 13.3 [574]	82.5 ± 14.8 [804]
US: Number of cysts in each kidney	36.6 ± 46.7 [220]	[< 10]	37.4 ± 62.5 [22]	30.1 ± 36.9 [33]	26.8 ± 21.7 [36]	40.2 ± 45.5 [53]	43.4 ± 56.8 [68]
eGFR	40.8 ± 24.4 [2478]	102.0 ± 23.9 [46]	77.4 ± 12.6 [303]	59.1 ± 11.8 [353]	46.3 ± 11.6 [466]	32.4 ± 7.9 [586]	16.0 ± 8.5 [724]
Height	164.8 ± 9.4 [2587]	164.4 ± 8.7 [46]	166.0 ± 9.4 [305]	165.7 ± 8.7 [353]	165.9 ± 9.2 [468]	164.4 ± 9.2 [590]	163.7 ± 9.8 [825]
γ-GTP	45.9 ± 61.4 [1923]	39.5 ± 57.5 [41]	35.8 ± 33.2 [256]	45.7 ± 59.5 [299]	52.5 ± 70.3 [396]	47.2 ± 60.2 [463]	45.4 ± 67.0 [468]
Creatinine	2.2 ± 2.4 [2467]	0.9 ± 1.7 [46]	0.8 ± 0.3 [301]	1.0 ± 0.6 [352]	1.3 ± 0.7 [466]	1.7 ± 0.4 [583]	4.4 ± 3.5 [719]
Systolic blood pressure	133.4 ± 17.0 [2532]	127.7 ± 12.6 [46]	129.1 ± 14.3 [296]	132.1 ± 15.0 [344]	133.0 ± 16.9 [460]	133.0 ± 17.0 [577]	136.3 ± 18.3 [809]
Kidney volume	1929.3 ± 1297.4 [2303]	1079.4 ± 399.0 [44]	1324.4 ± 799.0 [299]	1623.1 ± 1100.8 [338]	1865.0 ± 1180.1 [459]	2046.6 ± 1187.2 [553]	2399.0 ± 1588.4 [610]
Waist circumference	84.3 ± 10.5 [1409]	79.5 ± 11.4 [26]	81.3 ± 10.3 [187]	82.2 ± 9.1 [186]	84.8 ± 10.6 [268]	84.6 ± 10.2 [318]	86.2 ± 10.9 [424]
Weight	62.7 ± 13.0 [2587]	59.5 ± 13.4 [46]	62.3 ± 11.6 [305]	63.0 ± 11.7 [353]	64.7 ± 13.7 [468]	62.8 ± 12.3 [590]	61.8 ± 14.1 [825]
Valvular heart disease	238 (25.1%) [949]	< 10 (–%) [12]	26 (22.0%) [118]	27 (19.0%) [142]	40 (19.6%) [204]	64 (28.3%) [226]	79 (32.0%) [247]
Cerebral aneurysm clipping surgery	49 (5.5%) [887]	< 10 (–%) [11]	< 10 (–%) [77]	< 10 (–%) [86]	< 10 (–%) [130]	10 (5.6%) [180]	27 (6.7%) [403]
Cerebral aneurysm coil embolization	13 (1.5%) [887]	< 10 (–%) [11]	< 10 (–%) [77]	< 10 (–%) [86]	< 10 (–%) [131]	< 10 (–%) [180]	< 10 (–%) [402]
Dialysis therapy	217 (8.5%) [2568]	< 10 (–%) [46]	< 10 (–%) [302]	< 10 (–%) [349]	< 10 (–%) [464]	< 10 (–%) [585]	209 (25.4%) [822]
Erythropoietin	354 (13.9%) [2538]	< 10 (–%) [45]	< 10 (–%) [300]	< 10 (–%) [350]	< 10 (–%) [459]	13 (2.2%) [582]	330 (41.1%) [802]
Whether or not close relatives have the disease	1542 (86.1%) [1791]	34 (87.2%) [39]	229 (90.2%) [254]	249 (90.2%) [276]	304 (85.6%) [355]	380 (86.4%) [440]	346 (81.0%) [427]
Liver cyst puncture	55 (2.1%) [2565]	< 10 (–%) [46]	< 10 (–%) [303]	< 10 (–%) [350]	10 (2.1%) [468]	13 (2.2%) [586]	19 (2.3%) [812]
Hepatic cyst fenestration or partial resection	< 10 (–%) [898]	< 10 (–%) [11]	< 10 (–%) [80]	< 10 (–%) [87]	< 10 (–%) [135]	< 10 (–%) [181]	< 10 (–%) [404]
Liver cyst decannulation or removal	14 (0.7%) [2059]	< 10 (–%) [41]	< 10 (–%) [265]	< 10 (–%) [307]	< 10 (–%) [416]	< 10 (–%) [506]	< 10 (–%) [524]
Hepatic arterial embolization therapy	38 (1.5%) [2566]	< 10 (–%) [46]	< 10 (–%) [303]	< 10 (–%) [350]	11 (2.4%) [467]	< 10 (–%) [587]	11 (1.4%) [813]
Intracranial aneurysm	321 (18.7%) [1715]	< 10 (–%) [29]	34 (14.4%) [236]	50 (18.6%) [269]	62 (18.0%) [345]	81 (19.6%) [413]	94 (22.2%) [423]
Renal cyst puncture	36 (1.4%) [2562]	< 10 (–%) [46]	< 10 (–%) [303]	< 10 (–%) [349]	< 10 (–%) [466]	< 10 (–%) [589]	17 (2.1%) [809]
Renal cyst fenestration or removal	15 (0.6%) [2557]	< 10 (–%) [46]	< 10 (–%) [302]	< 10 (–%) [348]	< 10 (–%) [463]	< 10 (–%) [587]	10 (1.2%) [811]
Renal artery embolization therapy	29 (1.1%) [2568]	< 10 (–%) [46]	< 10 (–%) [303]	< 10 (–%) [350]	< 10 (–%) [466]	< 10 (–%) [589]	26 (3.2%) [814]
Kidney transplantation	< 10 (–%) [2567]	< 10 (–%) [46]	< 10 (–%) [301]	< 10 (–%) [349]	< 10 (–%) [466]	< 10 (–%) [586]	< 10 (–%) [819]
Liver transplantation	< 10 (–%) [823]	– [< 10]	< 10 (–%) [62]	< 10 (–%) [72]	< 10 (–%) [116]	< 10 (–%) [166]	< 10 (–%) [398]
Kidney volume 750mL or more and kidney volume growth rate 5% or more per year	1909 (86.5%) [2208]	42 (95.5%) [44]	282 (95.3%) [296]	319 (94.9%) [336]	400 (90.7%) [441]	437 (82.9%) [527]	429 (76.1%) [564]
Partial hepatectomy	< 10 (–%) [2058]	< 10 (–%) [40]	< 10 (–%) [265]	< 10 (–%) [306]	< 10 (–%) [416]	< 10 (–%) [506]	< 10 (–%) [525]
Partial hepatectomy/liver transplantation	< 10 (–%) [66]	– [< 10]	< 10 (–%) [14]	< 10 (–%) [13]	< 10 (–%) [15]	< 10 (–%) [14]	– [< 10]
Tolvaptan	469 (18.3%) [2559]	< 10 (–%) [46]	61 (20.1%) [303]	65 (18.6%) [349]	99 (21.2%) [466]	129 (22.1%) [585]	111 (13.7%) [810]
Occult blood	630 (26.4%) [2382]	< 10 (–%) [43]	61 (20.2%) [302]	65 (18.8%) [346]	101 (22.1%) [458]	128 (22.3%) [573]	268 (40.6%) [660]
Valvular heart disease	212 (18.4%) [1151]	< 10 (–%) [17]	23 (17.0%) [135]	23 (12.9%) [178]	37 (16.0%) [231]	54 (18.9%) [286]	74 (24.3%) [304]
Nursing care certification	14 (70.0%) [20]	0 [0]	– [< 10]	– [< 10]	– [< 10]	– [< 10]	– [< 10]
Colon diverticulum	132 (16.7%) [789]	< 10 (–%) [14]	12 (11.4%) [105]	16 (12.1%) [132]	33 (21.2%) [156]	21 (11.7%) [179]	49 (24.1%) [203]
Left cyst abundance	65 (100.0%) [65]	– [< 10]	14 (100.0%) [14]	14 (100.0%) [14]	15 (100.0%) [15]	14 (100.0%) [14]	– [< 10]
Right cyst abundance	65 (100.0%) [65]	– [< 10]	14 (100.0%) [14]	14 (100.0%) [14]	14 (100.0%) [14]	14 (100.0%) [14]	– [< 10]
Ductal plate abnormality	– [< 10]	0 [0]	0 [0]	0 [0]	0 [0]	0 [0]	– [< 10]
Typical image findings of unclear corticomedullary boundary, swelling, and high brightness	– [< 10]	0 [0]	0 [0]	– [< 10]	– [< 10]	0 [0]	– [< 10]
Liver cyst	1875 (88.6%) [2117]	30 (69.8%) [43]	246 (88.2%) [279]	284 (88.5%) [321]	382 (88.6%) [431]	461 (89.2%) [517]	472 (89.7%) [526]
Liver fibrosis	– [< 10]	0 [0]	0 [0]	0 [0]	– [< 10]	0 [0]	– [< 10]
High Blood Pressure	2132 (82.8%) [2574]	22 (47.8%) [46]	205 (67.4%) [304]	255 (72.4%) [352]	386 (82.8%) [466]	526 (89.6%) [587]	738 (90.1%) [819]
Infectious diseases (cyst infections, urinary tract infections)	376 (14.8%) [2533]	< 10 (–%) [45]	41 (13.7%) [300]	56 (16.1%) [348]	72 (15.7%) [460]	65 (11.3%) [576]	138 (17.2%) [804]
Cerebral aneurysm	311 (17.8%) [1751]	< 10 (–%) [33]	33 (13.8%) [239]	48 (17.4%) [276]	61 (17.2%) [354]	74 (17.5%) [424]	95 (22.4%) [425]
Cerebral hemorrhage	153 (6.0%) [2551]	< 10 (–%) [45]	14 (4.7%) [301]	24 (6.8%) [351]	31 (6.7%) [463]	33 (5.7%) [580]	50 (6.2%) [811]
Kidney pain (including urinary stones)	782 (31.7%) [2467]	11 (25.0%) [44]	90 (31.6%) [285]	104 (31.0%) [335]	148 (33.2%) [446]	162 (28.6%) [566]	267 (33.8%) [791]
Regular activities	1939 (92.5%) [2096]	39 (95.1%) [41]	264 (96.0%) [275]	299 (95.2%) [314]	392 (92.0%) [426]	478 (93.0%) [514]	467 (88.8%) [526]
Personal care	2042 (97.4%) [2096]	39 (95.1%) [41]	273 (99.3%) [275]	311 (98.4%) [316]	414 (97.4%) [425]	502 (98.0%) [512]	503 (95.4%) [527]
Anxiety/Depression	1789 (86.3%) [2074]	36 (87.8%) [41]	245 (90.4%) [271]	270 (86.3%) [313]	366 (87.1%) [420]	446 (87.6%) [509]	426 (81.9%) [520]
Degree of movement	2014 (95.9%) [2101]	40 (97.6%) [41]	270 (98.2%) [275]	312 (98.7%) [316]	407 (95.5%) [426]	496 (96.5%) [514]	489 (92.4%) [529]
Pain/discomfort	1666 (80.1%) [2080]	34 (85.0%) [40]	221 (81.5%) [271]	249 (79.8%) [312]	335 (79.4%) [422]	419 (82.8%) [506]	408 (77.1%) [529]
Macroscopic hematuria / cystic bleeding	615 (24.4%) [2517]	11 (23.9%) [46]	66 (22.2%) [297]	68 (19.5%) [349]	105 (22.8%) [461]	132 (23.0%) [574]	233 (29.5%) [790]
Gender: Male	1290 (49.9%) [2587]	21 (45.7%) [46]	138 (45.2%) [305]	166 (47.0%) [353]	246 (52.6%) [468]	303 (51.4%) [590]	416 (50.4%) [825]
Typical image findings in ultrasound images: unclear corticomedullary border, swelling, and high brightness (ARPKD only)	– [< 10]	0 [0]	0 [0]	0 [0]	0 [0]	0 [0]	– [< 10]
CKD severity A classification (A1 / A2 / A3)	383 (49.0%) / 211(27.0%) / 188 (24.0%) [782]	10 (90.9%) / <10 (–%) / <10 (–%) [11]	64 (81.0%) / 12(15.2%) / <10 (–%) [79]	59 (73.8%) / 16(20.0%) / <10 (–%) [80]	83 (64.3%) / 36(27.9%) / 10 (7.8%) [129]	98 (54.7%) / 57(31.8%) / 24 (13.4%) [179]	69 (22.7%) / 89(29.3%) / 146 (48.0%) [304]
CKD Severity G Classification (G1 / G2 / G3a / G3b / G4 / G5)	27 (3.0%) / 89 (10.0%) / 117 (13.2%) / 165(18.6%) / 214 (24.2%) / 274 (30.9%) [886]	< 10 (–%) / <10 (–%) / <10 (–%) / <10 (–%) / <10 (–%) / <10 (–%) [10]	17 (22.1%) / 56 (72.7%) / <10 (–%) / <10 (–%) / <10 (–%) / <10 (–%) [77]	< 10 (–%) / 25 (29.8%) / 49 (58.3%) / <10 (–%) / <10 (–%) / <10 (–%) [84]	< 10 (–%) / <10 (–%) / 54 (40.9%) / 60 (45.5%) / <10 (–%) / <10 (–%) [132]	< 10 (–%) / <10 (–%) / <10 (–%) / 90 (49.5%) / 79 (43.4%) / <10 (–%) [182]	< 10 (–%) / <10 (–%) / <10 (–%) / 12 (3.0%) / 124 (30.9%) / 264(65.8%) [401]
CKD Severity Classification Heat Map (Green / Yellow / Orange / Red)	425 (16.8%) / 389(15.4%) / 400 (15.8%) / 1315(52.0%) [2529]	38 (84.4%) / <10 (–%) / <10 (–%) / <10 (–%) [45]	233 (77.2%) / 58(19.2%) / <10 (–%) / <10 (–%) [302]	125 (35.8%) / 162(46.4%) / 37 (10.6%) / 25 (7.2%) [349]	25 (5.4%) / 140(30.2%) / 184 (39.7%) / 114 (24.6%) [463]	< 10 (–%) / 23 (3.9%) / 156 (26.8%) / 401(68.8%) [583]	< 10 (–%) / <10 (–%) / 14 (1.8%) / 771(98.0%) [787]
Protein (± / + / 2+ / 3+ / 4+)	1308 (60.2%) / 494(22.7%) / 267 (12.3%) / 87 (4.0%) / 18 (0.8%) [2174]	32 (80.0%) / <10 (–%) / <10 (–%) / <10 (–%) / <10 (–%) [40]	205 (81.0%) / 36(14.2%) / 10 (4.0%) / <10 (–%) / <10 (–%) [253]	227 (74.7%) / 55(18.1%) / 16 (5.3%) / <10 (–%) / <10 (–%) [304]	281(70.1%) / 75(18.7%) / 37 (9.2%) / <10 (–%) / <10 (–%) [401]	343 (65.3%) / 118(22.5%) / 54(10.3%) / <10 (–%) / <10 (–%) [525]	220 (33.8%) / 204(31.3%) / 148 (22.7%) / 64 (9.8%) / 15 (2.3%) [651]

Continuous variables are expressed as means and standard deviations. Categorical variables are expressed as n (%). Values for numbers of patients are shown in square brackets [].This statistical table was independently prepared and processed by the authors and differs from the table published by the Japanese Ministry of Health, Labour and Welfare. Following a request from the Ministry of Health, Labour and Welfare, all reported data are processed to ensure k‑anonymity (k = 10). For continuous variables, when the number of observations was 10 or fewer, statistical values were suppressed and shown as “[< 10]”. For categorical variables, when the number of true cases was 10 or fewer, the patient count was shown as “<10 (--%)” and the percentage was masked; when the total number of observations for a categorical variable was 10 or fewer, both the patient count and percentage were suppressed and shown as “– [< 10]”.

After hyper parameter tuning using 10-fold cross-validation, the parameters achieving the highest accuracy for each of the three models were as follows: for the random forest, the mtry was 35, the trees 500, and the min_n was 40; for the linear support vector machine, the cost was 0. 000977, and the margin was 0.10; and for the naive Bayes model, the smoothness was 1.25, the laplace was 2.25, and the threshold was 1.00.

**Fig. 1:**
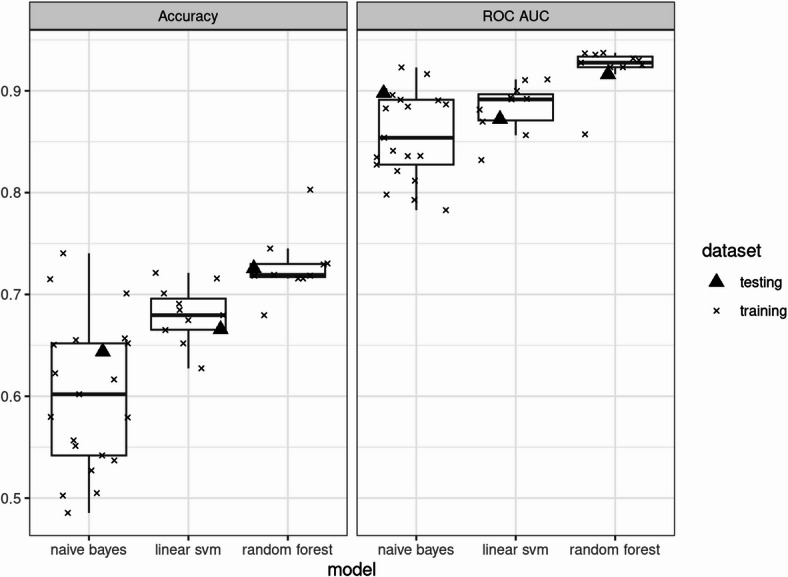
Comparison of accuracy and ROC-AUC among three methods in the training set. Boxplots display the distribution of accuracy (left) and ROC AUC (right) for all models. Each box represents the interquartile range (IQR), with the horizontal line indicating the median. Whiskers extend to 1.5 times the IQR. Individual data points are overlaid using jitter to visualize the distribution of data within each group.

Using these parameters, the final model for each method was constructed. Figure 1 illustrates the distribution of the evaluation results for the training set and the testing set in the final model. Among the models, random forest demonstrated the highest performance, followed by the linear support vector machine and the naive Bayes model.

**Fig. 2:**
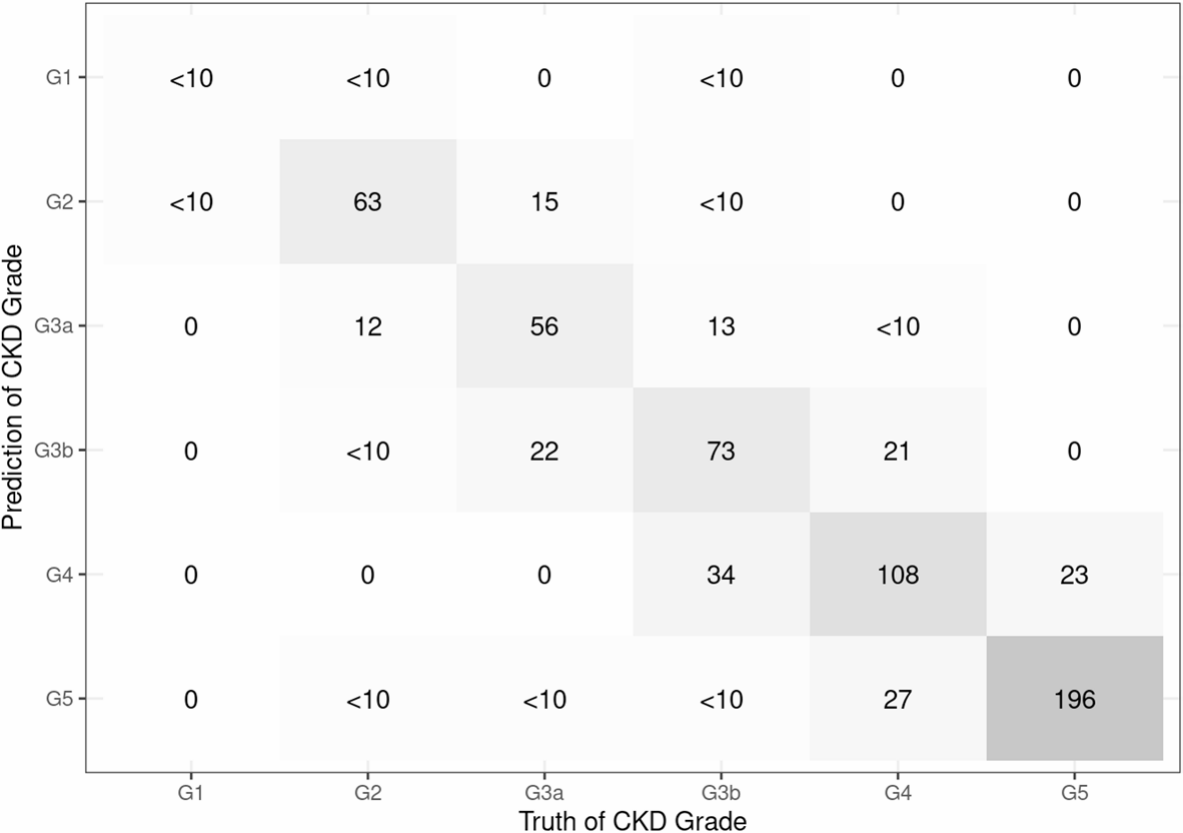
Confusion matrix of the random forest model in the testing set. The matrix displays the predicted labels versus the actual labels. Each cell represents the number of patients classified into the respective category. The diagonal values indicate correct classifications, while off-diagonal values represent misclassifications. Following a request from the Ministry of Health, Labour and Welfare, all reported data are processed to ensure k‑anonymity (k = 10); values were masked with the label “<10” when fewer than 10 patients corresponded to the data. All figures (Figs. 2, 3 and 4) apply the same masking rule.

**Fig. 3:**
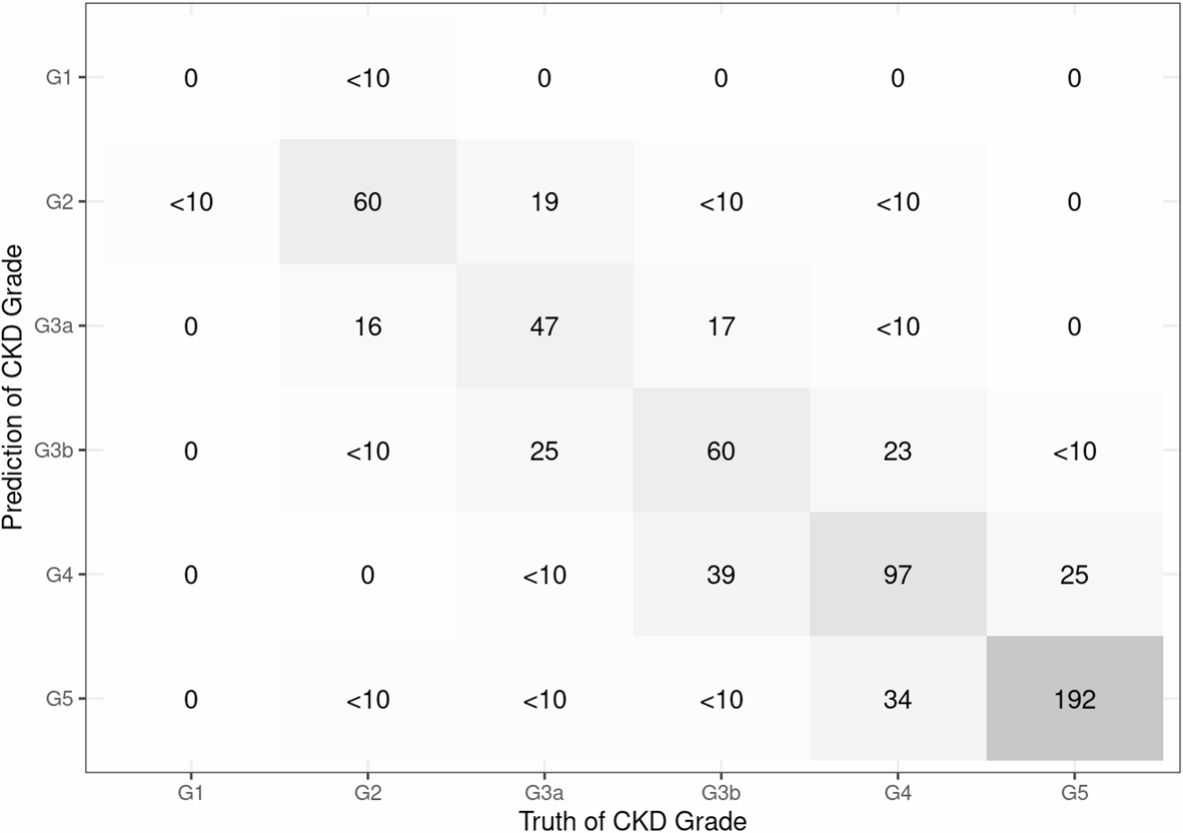
Confusion matrix of the support vector machine model in the testing set.

**Fig. 4:**
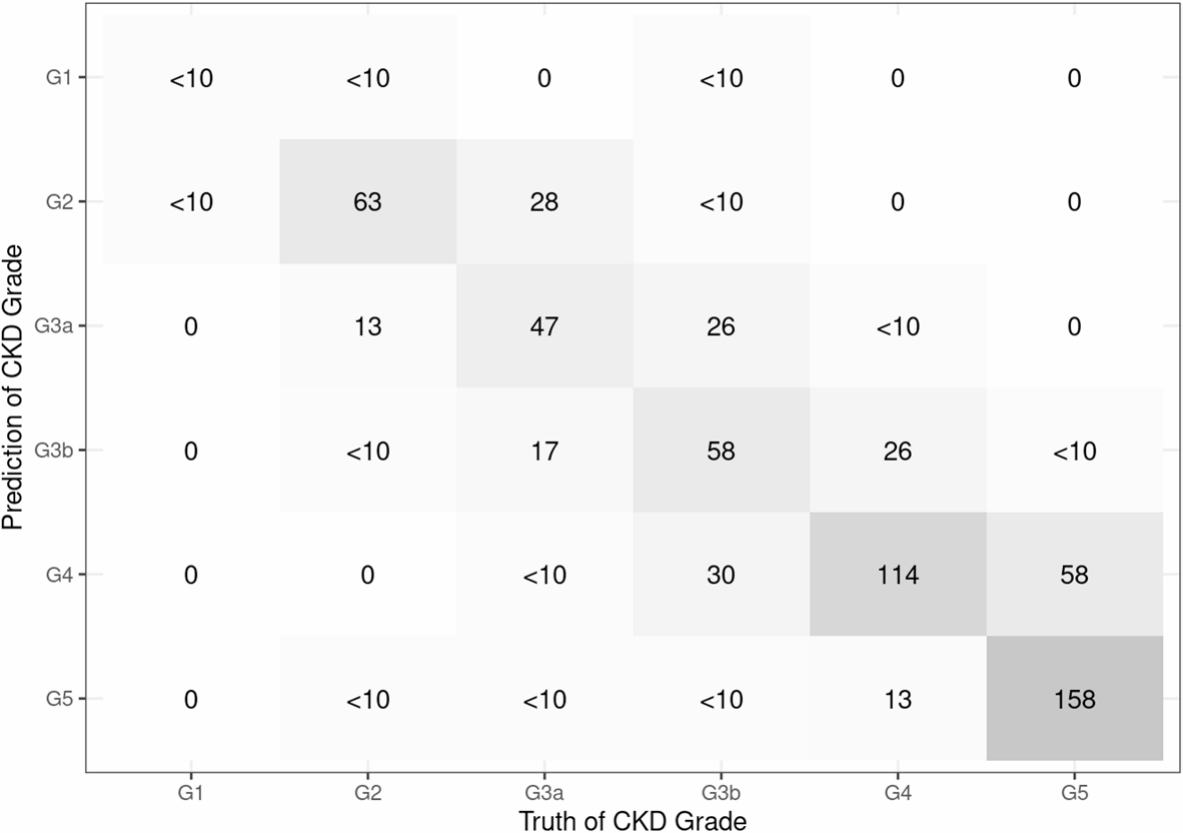
Confusion matrix of the naïve bayes model in the testing set.

**Fig. 5:**
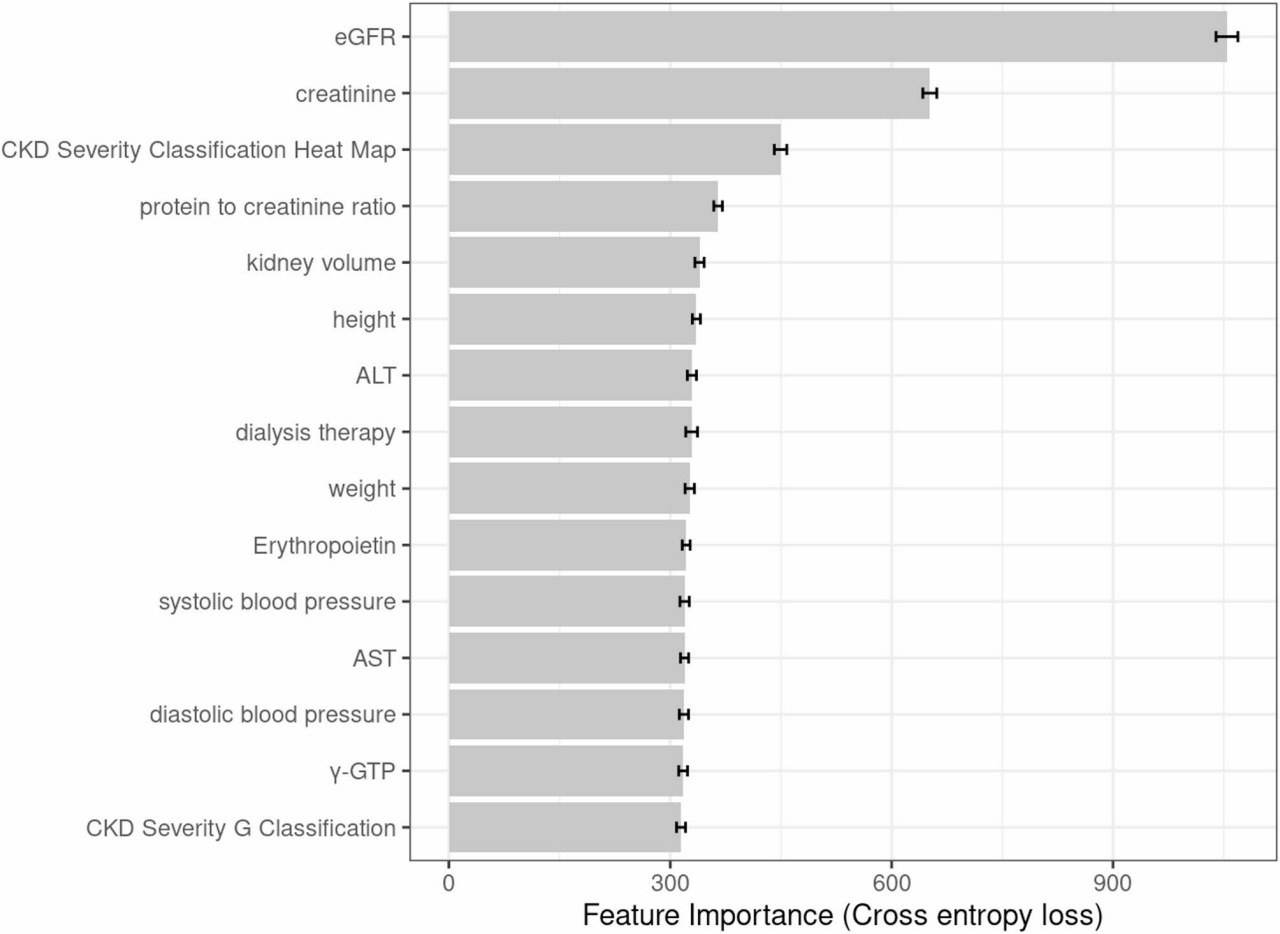
Feature importance of the random forest model (top 15). Bars represent the mean values of cross entropy loss after permutations. Error bars indicate standard deviation.

**Fig. 6:**
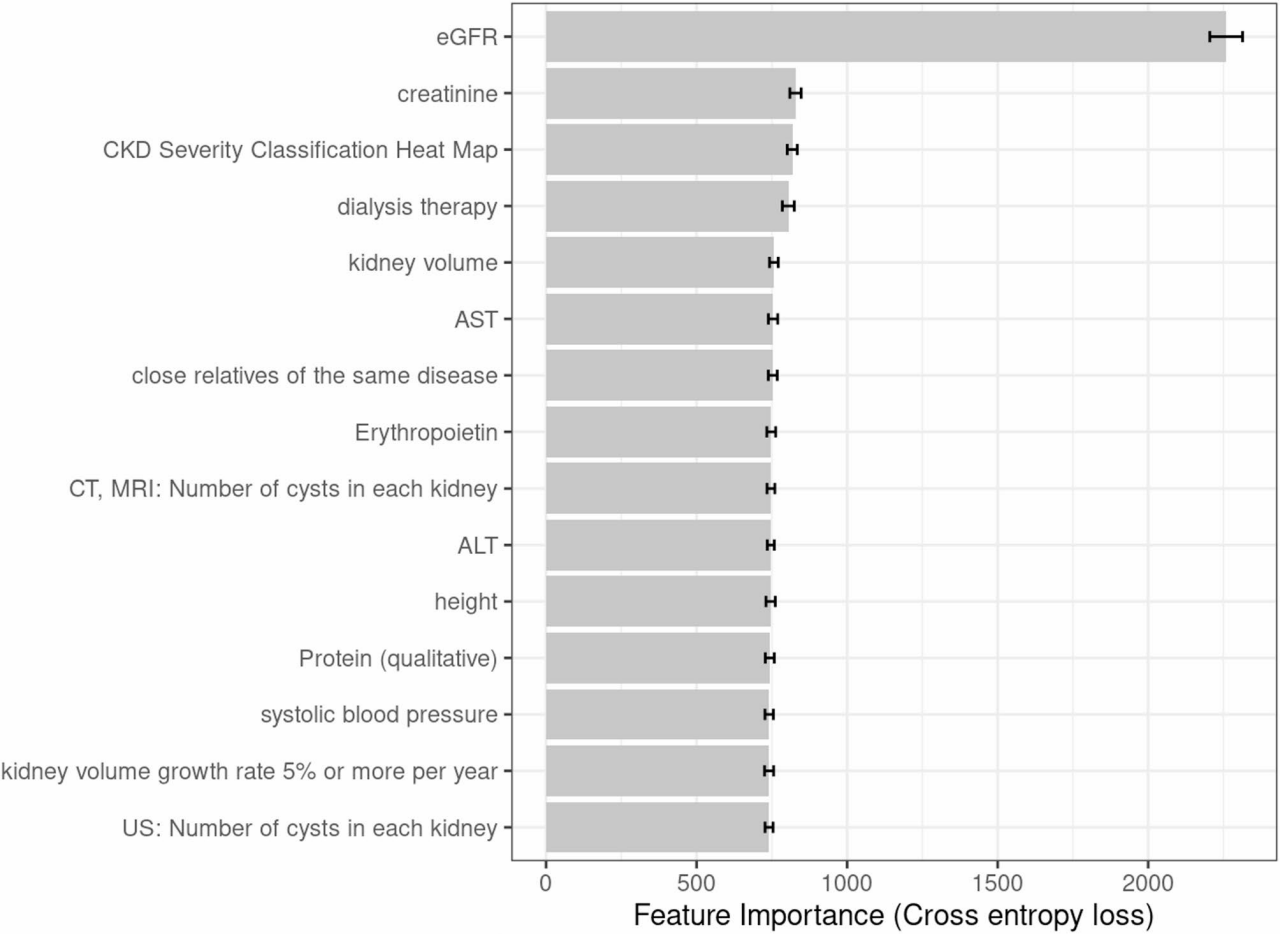
Feature importance of the linear support vector machine model (top 15). Bars represent the mean values of cross entropy loss after permutations. Error bars indicate standard deviation.

**Fig. 7:**
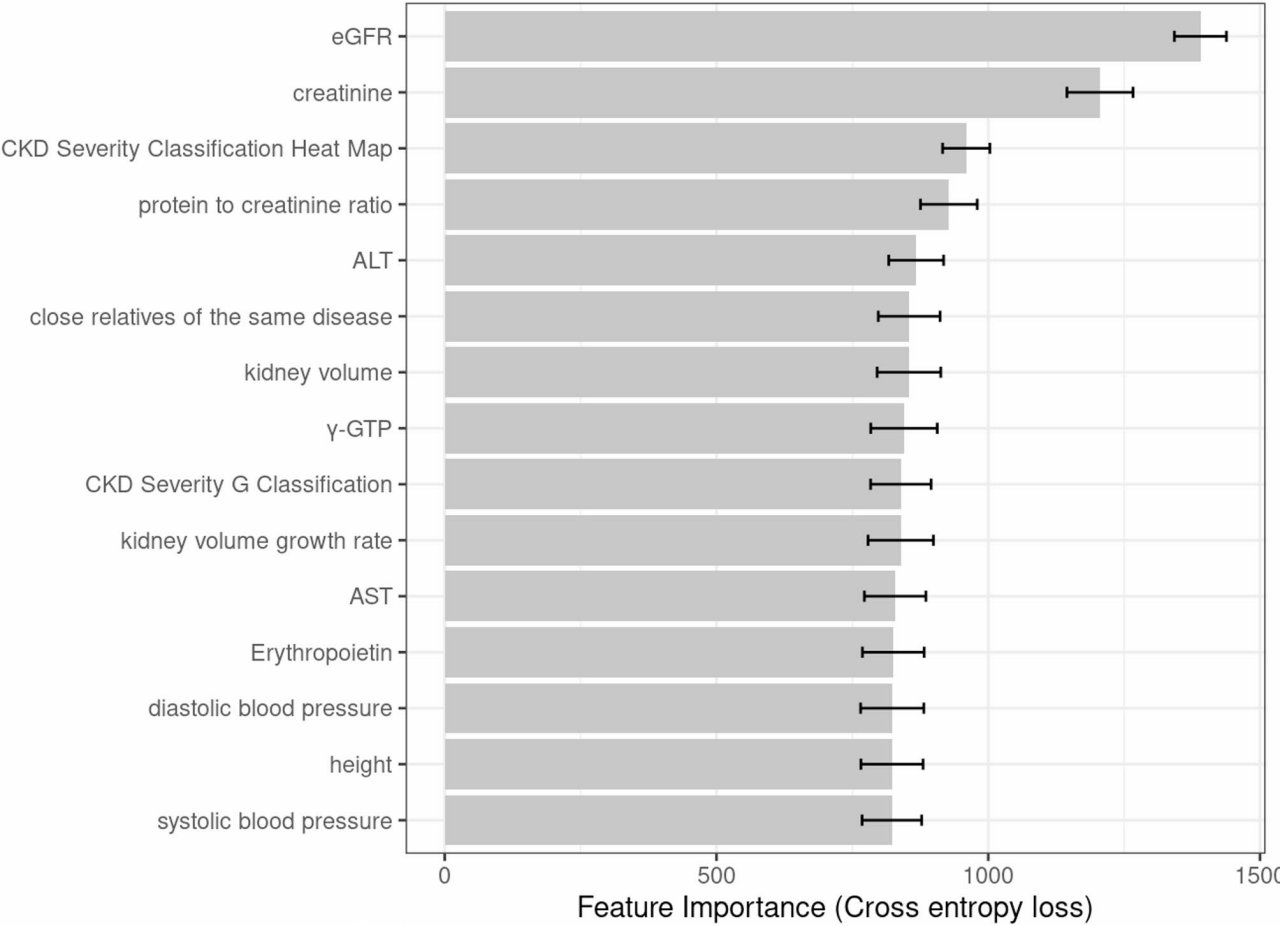
Feature importance of the naïve bayes model (top 15). Bars represent the mean values of cross entropy loss after permutations. Error bars indicate standard deviation.

The detailed evaluation results of the models on the testing set are presented in the confusion matrix shown in Figs. 2, 3 and 4. The confusion matrix for multi-class classification provides a comprehensive summary of the model’s predictions by comparing the true labels with the predicted labels across all classes. The most important explanatory variables for each model are depicted in Figs. 5, 6 and 7. Comparing the performance of the machine learning models on the testing set, the random forest model had an accuracy of 72.6%, while the linear support vector machine had an accuracy of 66.6%. The top 10 feature importance items in the random forest model were eGFR, serum creatinine, CKD heat map, protein to creatinine ratio, total kidney volume, height, alanine aminotransferase, weight, ESA drugs, and systolic blood pressure.

## Discussion

This study identified key factors and developed machine learning models to predict future CKD stages in patients with ADPKD. Among the models tested, the random forest model achieved the highest predictive accuracy on the test set. Feature importance analysis highlighted that, in addition to renal function markers such as eGFR and serum creatinine, CKD heat map and total kidney volume were also significant predictors of CKD stage.

Chronic kidney disease (CKD), including ADPKD, is a lifelong condition that requires personalized care to address patients’ diverse needs. The 2024 “Kidney Disease: Improving Global Outcomes” conference emphasized the need to move beyond a standardized approach to CKD management in favor of individualized treatment strategies^22^. Personalized medicine, which aligns with patient-centered care^23–26^, tailors treatments to individual characteristics^27^, a concept further enhanced by recent advancements in artificial intelligence and machine learning^5, 29, 30^.

Given the complex and heterogeneous nature of ADPKD, we hypothesized that machine learning could help address its variability. To explore this, we applied machine learning techniques to data from a Japanese nationwide registry of ADPKD patients. Machine learning’s ability to reduce dimensionality allows for ranking predictive variables in multifactorial chronic diseases^31^. Feature importance analysis using the random forest model identified the top 5 variables, so-called machine learning score^5^, eGFR, serum creatinine, CKD heat map, urinary protein, and total kidney volume as key predictors of CKD stage (Fig. 5).

The random forest, being a nonlinear model, is capable of capturing complex interactions, while the linear support vector machine is restricted to representing linear relationships. These findings suggest that nonlinear models are more effective for predicting renal prognosis. The naive Bayes model, which is a relatively simple approach assuming independence among explanatory variables and predicting outcomes by multiplying probabilities of occurrence, outperformed the linear support vector machine. These results indicate that a certain degree of independence exists among the explanatory variables, and in many instances, the variables exert minimal influence on one another.

Many factors influencing chronic diseases exhibit nonlinear relationships rather than simple linear correlations. Chronic diseases are often affected by complex interactions between genetic, environmental, lifestyle, and biological factors, which do not always follow a straightforward cause-and-effect pattern. Machine learning is particularly useful for analyzing chronic disease risk because it can model these nonlinear relationships more effectively than traditional linear statistical methods. In this study, random forest was the most useful of the three machine learning methods investigated, as it is the most tolerant of nonlinearity.

Across all models, predictive accuracy tended to be higher for stages G4/G5, whereas lower accuracy was observed for stages G2/G3. It is known that changes in eGFR are steep in stage G4/G5^32^, and once deterioration occurs, recovery is difficult; therefore, changes in G4/G5 are considered easier to predict. In contrast, changes in stages G2/G3 are more gradual, and with annual observations such as those used in the present study, early signs of change are difficult to capture, resulting in lower predictive accuracy for G2/G3. For prognostic prediction in stages G2/G3, data with more frequent measurement points will likely be required.

This study developed a prognostic prediction model for ADPKD that does not rely on genetic testing and can be applied in routine clinical practice. Although some explanatory variables were derived from imaging assessments made by the attending physician (e.g., the number of cysts), their importance was relatively small, suggesting that accurate prediction is feasible without dependence on MRI findings. Conventional prognostic models for ADPKD have often relied on costly genetic or MRI examinations; therefore, the present approach is expected to serve as a practical clinical decision‑support tool in daily clinical settings.

### Limitations

This study has several limitations. First, as an observational study, it does not allow for the establishment of causal relationships. Second, because the database used in this study contains information exclusively from Japanese individuals, the findings are subject to ethnic bias and may have limited generalizability to other populations. Third, the data were based on questionnaires completed by physicians assessing patients for intractable disease certification, which may introduce reporting biases. Fourth, since ADPKD is a lifelong chronic disease, long-term follow-up studies are needed to better understand its progression.

## Conclusions

Machine learning, particularly random forest, provides a robust approach for predicting CKD stages in patients with ADPKD by accounting for nonlinear variable relationships. These findings emphasize machine learning’s potential in personalized CKD management and highlight the need for individualized treatment approaches.

## Data Availability

The data that support the findings of this study are available from the Ministry of Health, Labour and Welfare of Japan but restrictions apply to the availability of these data, which were used under license for the current study, and so are not publicly available. Data are however available from the Ministry of Health, Labour and Welfare of Japan upon reasonable request and with permission. For inquiries regarding data availability, please contact the corresponding author, Satoru Muto.
